# Chest compression synchronized ventilation during prolonged experimental cardiopulmonary resuscitation improves oxygenation but may cause pneumothoraces

**DOI:** 10.1016/j.resplu.2025.100918

**Published:** 2025-02-28

**Authors:** Jukka Kopra, Lassi Mehtonen, Merja Laitinen, Erik Litonius, Oiva Arvola, Robert Östman, Juho A. Heinonen, Markus B. Skrifvars, Pirkka T. Pekkarinen

**Affiliations:** a Department of Emergency Care and Services, University of Helsinki and Helsinki University Hospital, Paciuksenkatu 1, 00290 Helsinki, Finland; b University of Latvia, Faculty of Medicine, Jelgavas street 3-330, LV – 1004, Riga, Latvia; c VetCT Teleconsulting – Teleradiology Small Animal Team, Helsinki, Finland; d Division of Anaesthesiology, Department of Anaesthesiology, Intensive Care and Pain Medicine, University of Helsinki and Helsinki University Hospital, Haartmaninkatu 4, 00290 Helsinki, Finland; e Department of Anaesthesiology and Intensive Care Medicine and Centre for Prehospital Emergency Care and Emergency Medicine, Päijät-Häme Central Hospital, Keskussairaalankatu 7, 15850 Lahti, Finland; f Division of Intensive Care, Department of Anaesthesiology, Intensive Care and Pain Medicine, University of Helsinki and Helsinki University Hospital, Haartmaninkatu 4, 00290 Helsinki, Finland

**Keywords:** Cardiac arrest, Ventilation, Arterial oxygen pressure, Mechanical chest compression, Mechanical ventilation, Computed tomography

## Abstract

**Background:**

Chest compression synchronized ventilation (CCSV) has been proposed to provide superior ventilation and haemodynamics during cardiac arrest (CA) compared to conventional asynchronous ventilation and compressions. We compared arterial gas exchange, pH, lactate levels and haemodynamics between CCSV and manual asynchronous ventilation during prolonged experimental CA.

**Methods:**

We randomized 30 pigs (weight ca. 55 kg) to receive CCSV with a MEDUMAT Standard^2^ ventilator or a manual bag valve targeting 10–12 ventilations per minute. Chest compressions were provided with a Lucas® 2 device. Arterial samples were drawn every 5 min and monitoring was recorded continuously. The animals underwent chest CT scans after death.

**Results:**

The median intra-arrest arterial blood gas results for CCSV were PaO_2_ = 490 (86–570) mmHg, PaCO_2_ = 20 (10–35) mmHg and pH = 7.39 (7.19–7.53). In the manual ventilation group, the results were PaO_2_ = 304 (109–379), PaCO_2_ = 36 (28–47) and pH = 7.24 (7.12–7.34). The oxygen levels were significantly higher in the CCSV group compared to a linear mixed model (*p* = 0.046). The differences in CO_2_ and pH levels were not statistically significant. The minute volumes and positive end-expiratory pressures were higher in the CCSV (18.0 [15.3–19.8] l/min; 32.6 [29.2–35.6] cmH_2_O) group compared to the control group (5.7 [4.9–7.0] l/min; 2.8 [1.8–4.1] cmH_2_O). The CCSV group had 12 pneumothoraces compared to 3 in the control group (*p* = 0.008).

**Conclusions:**

The CCSV protocol resulted in higher arterial oxygenation but more pneumothoraces.

The study was approved by the Finnish National Animal Experiment Board (ESAVI-26974-2023).

## Introduction

Refractory out-of-hospital cardiac arrest (OHCA) cases may require transport to hospital with ongoing CPR. Mechanical chest compression devices are recommended for use during transport to ensure rescuer safety and compression quality.^1^, ^2^ However, this could impair ventilatory performance,^3^, ^4^ possibly due to more severe dynamic air trapping and atelectasis formation.^5^ Many patients are hypoxic, hypercarbic and acidotic on hospital arrival despite the use of 100% oxygen and advanced airway control.^6^, ^7^, ^8^, ^9^, ^10^ Clinical data suggests that a high paO_2_ on eCPR initiation is associated with improved outcome.^11^ Therefore, ventilation strategies for patients undergoing prolonged mechanical CPR require more attention.^12^, ^13^

The chest compression synchronized ventilation (CCSV) protocol, developed by Kill et al.,^14^ has yielded promising preliminary experimental results of higher oxygenation, lower CO_2_ levels and higher pH after 13 min of cardiac arrest (CA). This method provides ventilation triggered by the rise in airway pressure caused by each inward chest compression. The chosen ventilation timing is assumed to prevent progressive atelectasis formation and atelectrauma caused by the cyclical recruitment–derecruitment. The rather high peak pressure of 60 mmHg is believed to be necessary to counteract the intrathoracic pressure created by chest compressions, but this may predispose to barotrauma in the hyperinflated lung areas. Thus far, no patient data from the CCSV method have been published in peer-reviewed journals.

We compared the CCSV protocol with conventional bag-valve ventilation in an animal model of prolonged defibrillation-resistant CA. We hypothesized that this ventilation method would provide better ventilation than the conventional protocol without hindering perfusion or causing major thoracic or lung tissue trauma. The primary endpoints were the levels of oxygen, CO_2_, pH and lactate in the femoral artery blood samples taken periodically during the CA. The secondary endpoints were the systolic and mean arterial pressures of a femoral artery cannula over time, the near-infrared spectroscopy (NIRS) values of the forehead over time and the frequency and severity of thoracic trauma findings in the postmortem CT scans.

## Materials and methods

This was an experimental animal study involving healthy landrace pigs (*n* = 30, both sexes) at the Laboratory Animal Centre, Large Animals Unit at the Viikki Campus of the University of Helsinki between November 2023 and December 2024. Ethical approval for the study was obtained from the Finnish National Animal Experiment Board (ESAVI-26974-2023). The study is reported in adherence with the ARRIVE guidelines (Supplementary Material 1).

### Preparation and monitoring

The fasted animals were premedicated with intramuscular injections of medetomidine (2 mg/kg) and racemic ketamine (10 mg/kg). An intravenous (IV) line was inserted into an ear vein, followed by anaesthesia induction with a bolus of IV propofol (1–1.5 mg/kg) and fentanyl (3–4 mcg/kg). Subsequently, endotracheal intubation was performed (Internal diameter 6.0–7.0 mm), and mechanical ventilation commenced using ∼21% oxygen (O_2_) minute ventilation targeting end-tidal carbon dioxide (EtCO_2_) of 5%. Cannulation of the internal jugular vein and femoral artery was performed with the Seldinger and open dissection techniques, respectively. A temporary pacemaker wire (Medtronic 5348 single-chamber temporary pacemaker, Soma Tech INTL, Bloomfield, CT, USA) was inserted via the jugular vein and floated into the right ventricle, with proper placement confirmed with ventricular pacing. Rocuronium (1 mg/kg) was given 1–3 min prior to inducing ventricular fibrillation (VF), followed by an additional 1 mg/kg dose during the CA in case of agonal breathing.

Arterial blood samples were analysed using a point-of-care device (i-STAT System, Abbott Laboratories, Princeton, NJ, USA). Haemodynamic and respiratory variables were monitored using an AS/3 Monitor with a M−COVX spirometry module (Datex-Ohmeda, GE Healthcare, Helsinki, Finland) and recorded with data collection software (iCentral® and S/5 iCollect® GE Healthcare, Helsinki, Finland), which automatically generated 10 s median values for the reported monitoring parameters.

A rectal temperature probe was inserted, and efforts were made to maintain a normal temperature of 38 °C. Cerebral oximetry was conducted using NIRS with sensors placed on the forehead and belly (INVOS TM5100C Cerebral Oximeter, Somanetics Inc., Troy, MI, USA).

### Experimental procedures

Baseline blood gas samples were collected from the central venous and arterial lines. During the CA phase, arterial blood samples were collected at 5-minute intervals and a venous sample from the central venous catheter at the 30 min time point.

**Fig. 1:**
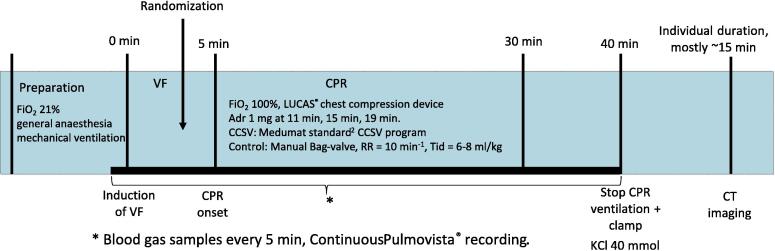
Timeline of the experimental protocol. Abbreviations: Adr, adrenaline; CCSV, chest compression synchronized ventilation; CPR, cardiopulmonary resuscitation; CT, computed tomography; FiO_2_, fraction of inhaled oxygen; KCl, potassium chloride; PEEP, positive end-expiratory pressure; RR, Respiratory rate, Tid, Tidal volume; VF, ventricular fibrillation.

Fig. 1 depicts the experiment timeline. After induction of VF using a 9 V direct current, ventilation and anaesthesia were stopped, and the pacing wire was removed. During the 5 min hand-off period, the animals were randomized to CCSV or manual ventilation with sealed opaque envelopes prepared 1:1 for each experiment week. The Lucas 2® compression device (Jolife AB/Stryker, Lund, Sweden) was set up, and the pig’s position was stabilized with a vacuum mattress and towels fitted inside free spaces between the Lucas arch and the pig’s sides. During CPR, ventilation was provided according to the assigned group protocol with 100% fraction of inspired oxygen (FiO_2_). The CCSV protocol was as follows: MEDUMAT Standard^2^ (respiratory rate [RR] = 100 min^−1^; inspiratory pressure [p_insp_] = 60 mmHg, trigger level 1–3, positive end-expiratory pressure 2–4 mbar, mechanical compression mode, Weinmann Emergency, Hamburg, Germany); for the manual bag-valve ventilation, RR = 10/min, tidal volume = 8–10 ml/kg, FiO_2_ = 100%, GE resuscitation bag (GE Healthcare, Helsinki, Finland), that had an expiratory valve preventing entrainment of air^15^ and equipped with a PEEP valve set to 0 cmH2O PEEP. The bag-valve was connected to a fresh flow of oxygen of 15 L/minute and equipped with an oxygen reservoir bag attached with an intake reservoir valve. Spirometry monitoring was available to the person providing manual ventilations as a guide with regards to tidal volumes and ventilation frequency. Adrenaline was administered intravenously at specified time points (11, 15 and 19 min) chosen to mimic the guideline-recommended^16^ time point after the third rhythm analysis. At the experiment’s end after 35 min of advanced life support, the animals were euthanized with a 40 mmol dose of potassium chloride, and the intubation tubes were clamped after a full manual ventilation. The CT scans were collected in the prone position, typically 15 min after death.

### Data processing

The 10 s median values of monitoring parameters generated by iCollect software were further processed by aggregating a median of six values around 5 min interval points. These median values were used for statistical analysis. Supplementary spirometry data analyses recorded by the MEDUMAT Standard^2^ ventilator (CCSV group only) were kindly provided by Weinmann (Supplementary material 2).

The CT scans were evaluated by a board-certified veterinary radiologist blinded to the intervention group with Osirix MD version 12.5.0 (VetCT, Cambridge, UK). The Hounsfield unit (HU) values were measured from 10 lung slices. The first slice was chosen from the most cranial aspect of the lungs, where both the left and right lobes appear on the same transverse slice. The most caudal slice was chosen at the level where it was still possible to divide both hemithoraces into 2–3 segments, but the accessory lobe was no longer visible. Eight evenly spaced slices were then defined from between these two.

**Fig. 2:**
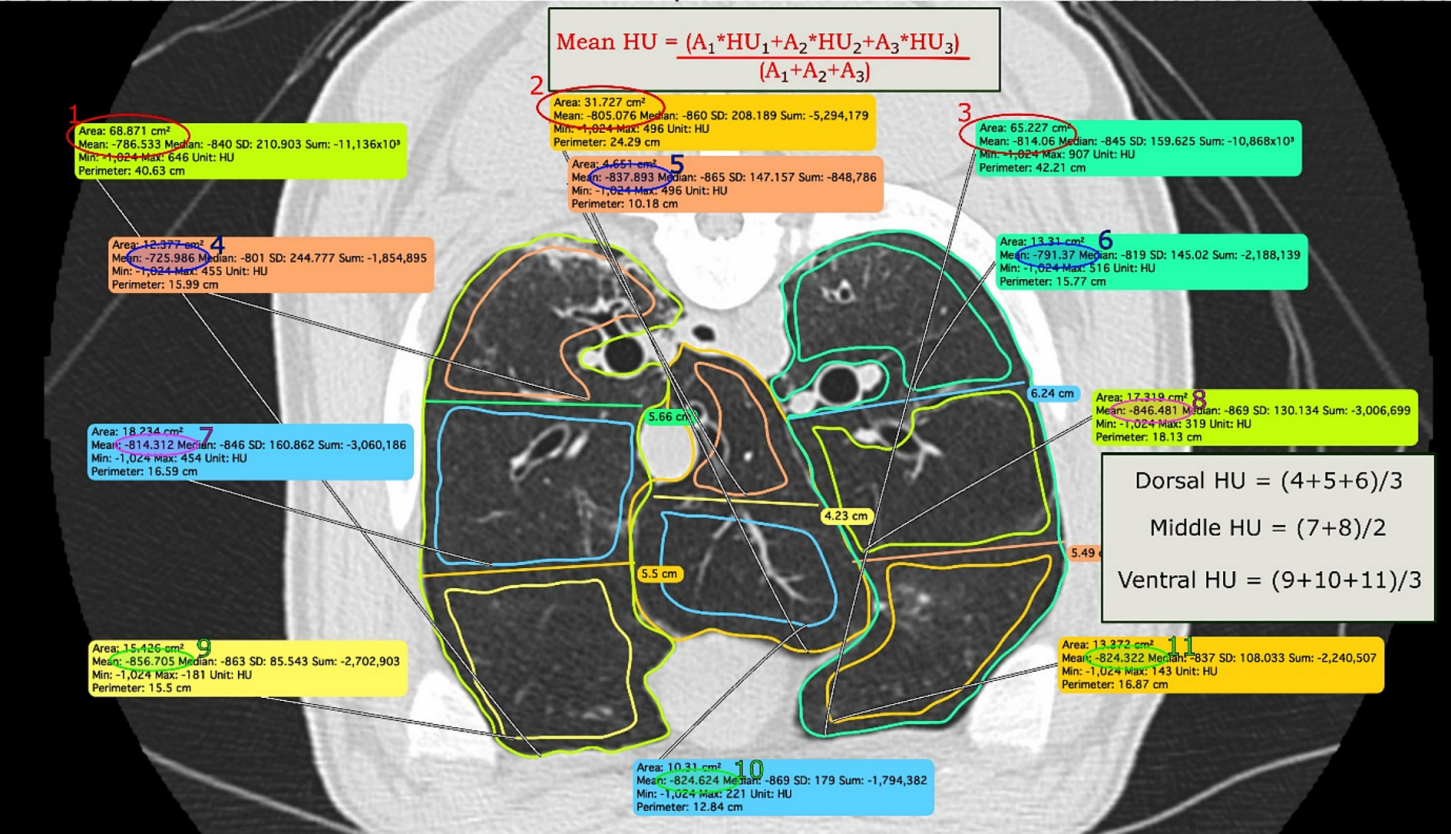
Schematic illustration of the analysis and calculations of a single computed tomography slice for the mean Hounsfield unit (HU) values and HU gradient values. The lung parenchyma of the hemithoraces and accessory lobes was delineated manually, and the mean HU value was assigned automatically to each area by the Osirix software. The mean HU values were then normalized to the according area, summed and divided by the total number of areas to calculate the average HU of the overall lung. The hemithoraces were also divided into 2–3 ventrodorsal segments of equal height, and representative HU values of these segments were averaged for the dorsal, middle and ventral HU values without normalization to the according area.

Fig. 2 illustrates the HU measurements and analysis process. The approximate size of the caudal pulmonary vessels was also visually estimated and compared. The relative size of the pulmonary vein was compared to the pulmonary artery (PV:PA) and the bronchi between the vessels. The sizes were compared using a scale of smaller – equal – larger. For statistical purposes, the subjects were grouped into PV > PA and PV ≤ PA classes. A finding of PV > PA is interpreted as a sign of pulmonary vein congestion due to failed left ventricle performance.

### Statistical analysis and sample size

Due to the non-parametric nature of most variables, the data are reported as medians and interquartile ranges (IQRs). Statistical significance for single comparisons was assessed using the Mann–Whitney *U* test. Multiple time point measurements were analysed using a linear mixed-effects model and a heterogeneous Toeplitz covariance matrix. The significant effects of the interventional group, time and the interaction term of time and the intervention group were assessed and reported as fixed effects (F). The figures were plotted with medians and IQRs. The statistics were calculated with IBM SPSS statistics software version 29.0.0.0.

For the sample size estimation, we anticipated a mean partial pressure of oxygen (PaO_2_) level of 600 ± 300 mmHg in the CCSV group and 250 mmHg in the control group, deriving the mean values from Kill et al. (2014). With an alpha of 0.05 and a power of 0.8, a sample size of 12 was recommended. We decided on a sample size of 15 per group to compensate for possible exclusions due to technical problems.

## Results

Fifteen animals were randomized to the CCSV group and 15 to the control group. One subject in the CCSV group was excluded from the analysis due to failure to provide ventilation with the MEDUMAT Standard^2^ ventilator, which arose from a water seal formed in a hosing valve.

No cases of return of spontaneous circulation were encountered. Most subjects deteriorated into asystole during the CPR. Three subjects in the CCSV group and five subjects in the control group persisted in VF until the end.

The groups’ baseline measurements are displayed in Table 1, with no reportable between-group differences. Table 2 presents the intra-arrest variables in which measurements remained rather constant throughout the CPR. We report high mean expiratory pressure levels in the CCSV group, very high minute volume levels and lower than expected ventilatory rates of (median 65.5/min).

**Table 1:** Baseline characteristics of the intervention groups.

	CCSV	Control	p-value
	Median (IQR)	Median (IQR)	(MWU)
Weight (kg)	55 (49.8–56.3)	54 (50–58)	0.63
Heart rate (bpm)	124 (114–139)	123 (115–140)	0.72
FiO_2_ (%)	23.3 (22.5–25.9)	23.6 (22.1–24.1)	0.75
Peak airway pressure (cmH_2_O)	23.0 (21.2–24.7)	21.3 (20.5–23.8)	0.4
Tidal volume (ml)	504 (485–534)	501 (483–602)	0.75
Compliance (ml/cmH_2_O)	44.4 (36.5–48.1)	42.9 (40.1–48.9)	0.59
pH	7.55 (7.50–7.57)	7.53 (7.50–7.55)	0.48

Abbreviations: bpm, beats per minute; CCSV, chest compression synchronized ventilation; cmH_2_O, centimetres of water; FiO_2_, fraction of inspired oxygen; IQR, interquartile range; MWU, Mann–Whitney *U* test.

**Table 2:** Vital signs and physiologic variables during cardiopulmonary resuscitation.

	CCSV	Control	p-value (MWU)
	Median (IQR)	Median (IQR)	
Respiratory rate (min^−1^)	65.5 (60.0–73)	10.0 (9.0–11.0)	**<0.001**
FiO_2_ (%)	95.2 (94.2–95.9)	94.2 (93.4–94.9)	**<0.001**
Minute volume (litres)	18.0 (15.3–19.8)	5.7 (4.9–7.0)	**<0.001**
Expiratory tidal volume (ml)	235 (138–483)	533 (465–594)	**<0.001**
Peak pressure (cmH_2_O)	54.9 (51.5–60.7)	44.3 (35.1–60.1)	**<0.001**
Compliance (ml/cmH_2_O)	16.2 (13.1–26.1)	24.5 (16.9–31.8)	**0.001**
Mean expiratory pressure (cmH_2_O)	32.6 (29.2–35.6)	2.8 (1.8–4.1)	**<0.001**
Venous pH	7.06 (6.97–7.10)	7.03 (6.98–7.13)	0.85
Venous lactate (mmol/l)	12.7 (11.5–14.6)	13.6 (10.1–14.6)	0.56
PvO_2_ (mmHg)	20.6 (18.3–24.5)	21.9 (17.3–25.7)	0.85
PvCO_2_ (mmHg)	71.2 (64.0–85.7)	80.0 (71.5–87.5)	0.17

cmH_2_O, centimetres of water; CPR, cardiopulmonary resuscitation; FiO_2_, fraction of inspired oxygen; IQR, interquartile range; MWU, Mann–Whitney *U* test; PEEP, positive end-expiratory pressure; PvCO_2_, venous pressure of carbon dioxide; PvO_2_, venous pressure of oxygen.

### Arterial blood measurements

**Fig. 3:**
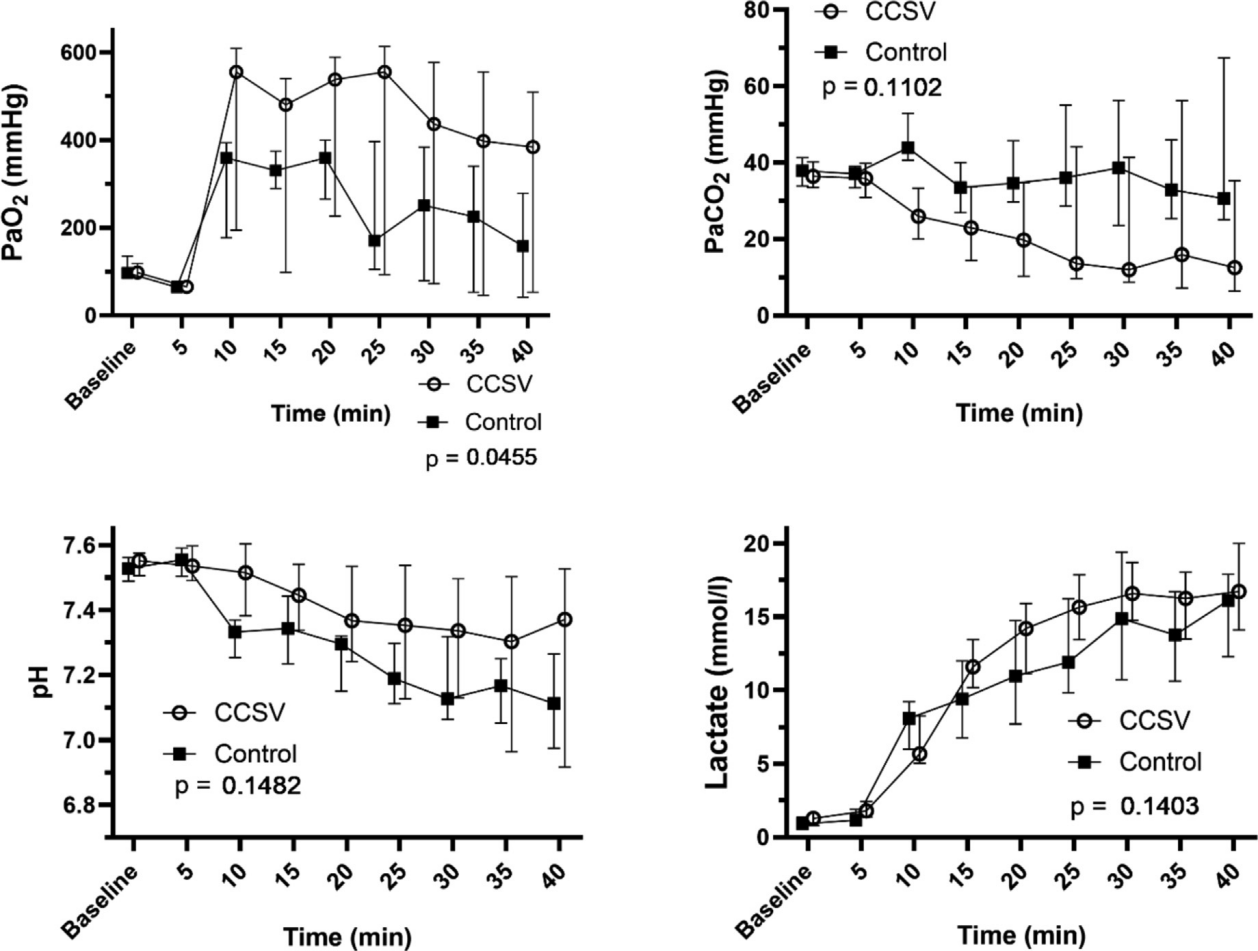
Arterial oxygen pressures (PaO_2_), carbon dioxide pressures (PaCO_2_), pH and lactate levels during experimental cardiopulmonary resuscitation shown as medians and interquartile ranges. The p-value is given for a linear mixed model between the groups. Abbreviation: CCSV, chest compression synchronized ventilation.

The arterial blood oxygen, CO_2_, pH and lactate results with corresponding p-values are shown in Fig. 3. The CCSV group’s arterial oxygen levels were significantly higher than those of the manual ventilation group throughout the experiment (*F* = 4.40, *p* = 0.048); the CO_2_, pH and lactate levels did not differ. The time × group interaction term was statistically significant for lactate (*F* = 3.28, *p* = 0.0015), and nonsignificant for PaO_2_ (*p* = 0.88), PaCO_2_ (*p* = 0.76) and pH (*p* = 0.42).

### Haemodynamics and end-tidal CO_2_

**Fig. 4:**
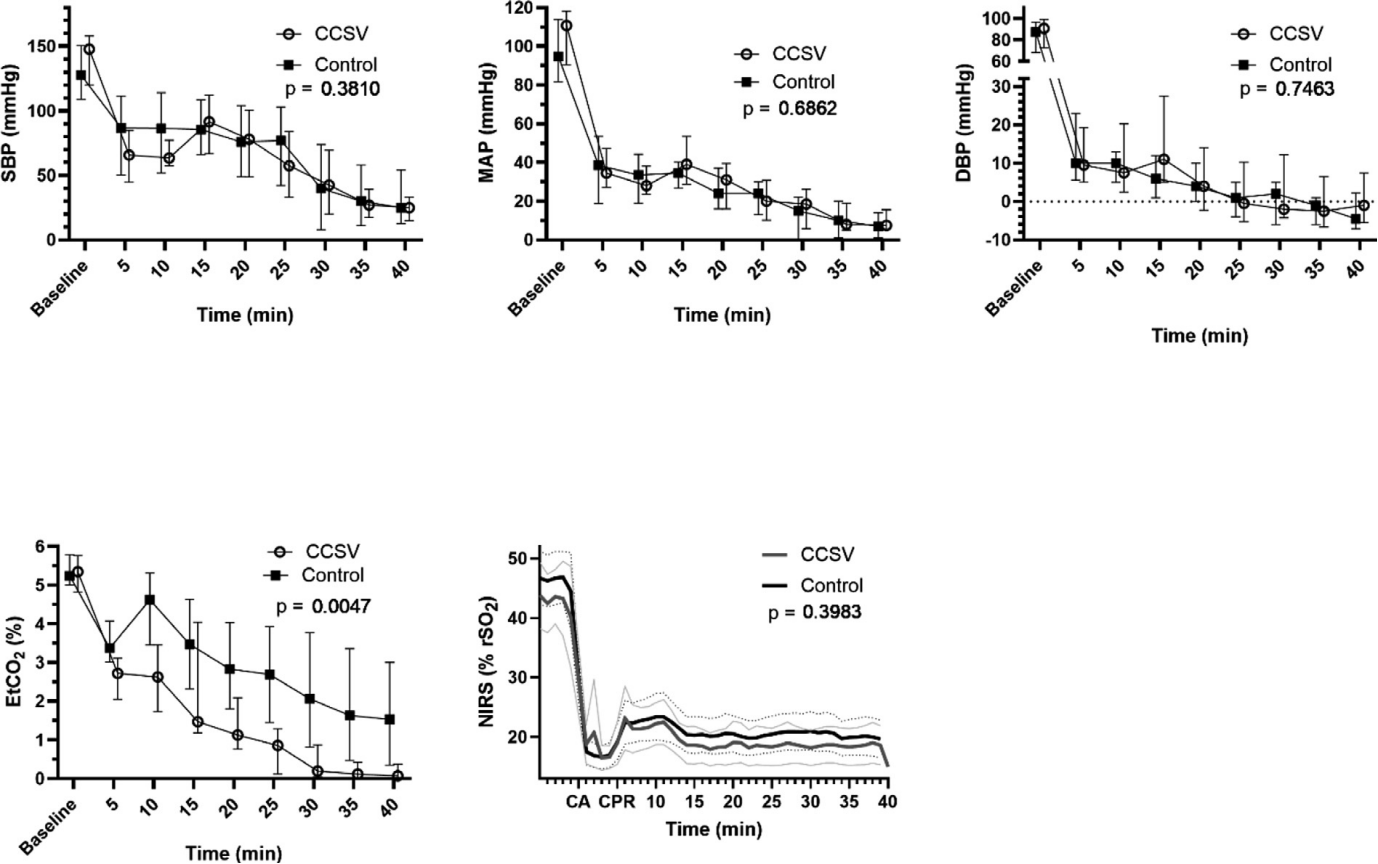
Systolic blood pressure (SBP), mean arterial pressure (MAP), diastolic blood pressures (DBP), end-tidal carbon dioxide (EtCO_2_) levels and near-infrared spectroscopy (NIRS) values during experimental cardiopulmonary resuscitation (CPR) shown as medians and interquartile ranges. The p-value is given for a linear mixed model between the groups. Abbreviations: CCSV, chest compression synchronized ventilation, CA, cardiac arrest.

Fig. 4 shows the systolic blood pressure levels, mean arterial pressure levels, EtCO_2_ levels and NIRS values with corresponding p-values. The CCSV group had lower EtCO_2_ levels throughout the CPR (*F* = 9.50, *p* = 0.0047), whereas the blood pressure and NIRS values were similar. The time × group interaction term was significant for EtCO_2_ (*F* = 3.17, *p* = 0.021) and nonsignificant for systolic blood pressure (SBP) (0.11), mean arterial pressure (MAP) (0.81) and NIRS (0.79).

### Postmortem computed tomography

Table 3 reports the CT findings. No statistically significant differences were found in the number of rib fractures, mean lung density of the lungs or regional mean densities in the ventral-to-dorsal axis. The CCSV group had significantly more pneumothoraces than the control group, with most being markedly severe and a handful even tensional. Lung volumes were lower in the CCSV group than in the manual ventilation group, but this was not significant (*p* = 0.077), likely due to the higher prevalence of pneumothoraces in the CCSV group. The pulmonary venous diameter more commonly exceeded the pulmonary arterial diameter (PV/PA > 1) in the control group than in the CCSV group (*p* = 0.002).

**Table 3:** Computed tomography findings after death.

	CCSV	Control	p-value
	Median (IQR)	Median (IQR)	MWU
	n = 14	n = 15	
Rib fractures (# broken ribs per subject)	5 (5–5)	5 (5–6)	0.65
Displaced fractures (# broken ribs per subject)	2.5 (2–3)	3 (2–4)	0.4
Mean density (HU)	-572 (-450 to -653)	-582 (-465 to -623)	0.91
Ventral lung density (HU)	-593(-460 to -719)	-705 (-605 to -785)	0.11
Middle lung density (HU)	-548(−417 to −672)	-652 (-502 to - 701)	0.33
Dorsal lung density (HU)	-487 (−393 to −641)	-508 (-362 to -585)	0.59
Pneumothoraces (mild/moderate/marked)	2/2/8	1/2/0	**0.008**
No pneumothorax	2	12	
Computed lung volume (l)	0.72 (0.48–1.3)	1.1 (0.99–1.2)	*0.077*
CT findings of venous congestion (PV/PA > 1)	3/15	11/13	**0.002**

Abbreviations: CT, computed tomography; CCSV, chest compression synchronized ventilation; IQR, interquartile range; MWU, Mann–Whitney *U* test; HU, Hounsfield unit; PV/PA, pulmonary vein/pulmonary artery diameter index.

## Discussion

### Key findings

In this experimental model of prolonged CPR, our findings suggest that the CCSV protocol provides higher arterial blood oxygenation throughout the 35 min of chest compressions compared to manual bag-valve ventilation. The minute volumes were threefold in the CCSV protocol even with suboptimal chest compression synchronization. We identified a significant number of marked pneumothoraces in the CCSV group. The possible relevance of these findings is unclear. It may be that pneumothoraces are more common in the pig after prolonged CPR. Nonetheless, this should be carefully looked at in clinical trials that implement the CCSV protocol in clinical practice.

### Relationship to previous studies

Accumulated evidence shows that intra-arrest arterial oxygen levels tend to be hypoxic, even with intubated patients, let alone with less definitive airway management.^6^, ^10^ Use of laryngeal tubes has been connected to more anoxic brain damage compared to endotracheal tubes. The current guideline-recommended ventilatory protocol is expert opinion level with very sparse patient data published,^12^ and clinical adherence is low.^17^ Problems with both manual and mechanical ventilation have been described.^13^, ^18^, ^19^

To our knowledge, there are four publications on the CCSV protocol in experimental settings and no published patient data, even though the protocol is already actively used in some centres. Kill et al. (2014), in their foundation work, reported superior oxygenation and CO_2_ clearance of arterial blood at 4 and 13 min time points of CA with the CCSV protocol (*n* = 8). Furthermore, they reported slightly enhanced haemodynamics compared to intermittent positive pressure ventilation (IPPV) (*n* = 8) and bilevel positive pressure ventilation (*n* = 8), leaving little concern for deleterious effects^20^, ^21^ on blood flow with the protocol’s high minute volumes. In their second study, Kill et al.^22^ compared three different pressure support levels and inspiration times of the CCSV protocol in a randomized sequential order of 4 min time periods within the same experiment (*n* = 12). The CPR was always started and finished with 4 min periods of IPPV protocol used as comparator phases. All the CCSV protocol adjustments displayed higher arterial oxygen content, and the higher pressure support levels displayed enhanced CO_2_ clearance compared to IPPV. The MAP levels were also higher in the CCSV protocol periods. However, the 4 min periods are, in our view, rather short for the gas exchange and haemodynamics to properly display a specific treatment effect witnessable amongst the typical deteriorating trend of CPR. Nevertheless, the deterioration was more pronounced after the final 4 min IPPV phase. Xu et al.^23^ compared four groups: CCSV vs. IPPV vs. both with aortic balloon occlusion (ABO) in a relatively short CPR model of 8 min. They also found enhanced oxygenation and CO_2_ clearance with CCSV compared to IPPV. The whole CCSV + ABO group achieved ROSC, a statistically significant finding compared with the IPPV-only group. Renz et al.^24^ published the results of ROSC and post-ROSC neuroinflammatory marker levels. They reported lower ROSC rates for CCSV (4/10) than for the other groups (8/10 and 9/10), hypothesizing that the CSSV protocol may have caused deleterious lung damage to the non-surviving subjects. No ventilatory or haemodynamic data for the CPR phase were published, but the neuroinflammatory marker levels were lower in the CCSV group than in the other groups.

These promising earlier studies prompted us to investigate the performance of the CCSV protocol in our established prolonged experimental model.^25^, ^26^ The high number of pneumothoraces compared to the control group and our previous studies was a surprising finding not suggested by previous CCSV studies. In this study, we used a “plug-and-play” approach with default CCSV settings. The results suggest that successful clinical use of CCSV may require active adjustment of CCSV ventilator settings during resuscitation. We were unable to display superior perfusion pressures, possibly partly due to the pneumothoraces, and the same applies to the nonstatistical differences in the PaCO_2_ and pH levels.

We present the PV/PA findings as an exploratory surrogate for the possible development of lung oedema and venous stasis. The control group had many more index-positive subjects, again possibly partly explained by the lower incidence of pneumothoraces. Intrathoracic pressure buildup possibly prohibits venous return and thus lung congestion. However, the control group also had much more consolidated lung tissue than the CCSV group, explaining the comparable mean HU values in the groups, despite minimal pneumothorax problems in the control group. The PV/PA index has been found to correlate with congestive heart pathology in canine and feline patients and, conversely, to correlate with pulmonary embolism in human and veterinary patients.^27^, ^28^, ^29^, ^30^, ^31^, ^32^ We presume that the PV/PA index does not react as much to Euler-Liljestrand mechanism as to pulmonary venous congestion due to left ventricle failure, or large pulmonary embolisms, where much of pulmonary vasculature is totally obstructed.

### Study strengths and limitations

Our study mimicked a defibrillation-resistant OHCA transported to hospital for further invasive procedures, in which ventilation performance is pronounced compared to shorter durations of CA. The study’s strength lies in the prolonged timeframe with multiple blood gas sampling, providing insight into this less charted area of CPR. The CCSV protocol maintained high oxygen levels, low CO_2_ and close to normal pH levels in many resuscitated subjects throughout the experiment, warranting optimistic expectations for future research. The EtCO_2_ curves displayed a close-to-total washout of CO_2_ in the exhaled gas mixture towards the later phase of CPR probably due to the almost threefold minute volume difference, and an unmeasurably low PaCO_2_ level was reached in a few subjects. We believe that in the context of CPR, hyperoxia is not as deleterious as after ROSC. In previous studies very high arterial oxygen pressures are not that common.^25^, ^26^, ^33^, ^34^

The somewhat wedge-shaped contour of the porcine thoracic cage and the unphysiological supine CPR positioning seem plausible explanations for the high incidence of pneumothoraces. These findings may not apply to humans in the same extent, though the CCSV protocol appeared to result in more pneumothorax formation than is some previous studies.^25^, ^26^ There was marked heterogeneity in our study outcome variables, downplaying the statistical power achieved. This was partly due to early waning perfusion pressures in some subjects. Halted or near halted perfusion disables the gas exchange, no matter the ventilation. The lack of invasive cerebral perfusion monitoring is an important limitation as the CCSV ventilatory pressures may have affected blood perfusion to the brain during the CPR. We did not, however, find any difference in brain oxygenation measured with NIRS.

Our spirometry monitor is not calibrated for the high respiratory rates of CCSV and after closer examination of the recorded data we noticed that the iCollect derived PEEP values resembled more mean expiratory pressures. The end-expiratory pressure levels from MEDUMAT Standard^2^ are given in Supplementary material 2. There was also discrepancy in respiratory rate results with MEDUMAT derived values reaching 86 (75–101) min^−1^. These discrepancies depict a hampered reliability of the iCollect data in the CCSV monitoring. In any case, we still report problems with achieving a high level of synchronization to the compressions, displayed by the lower than 100 min^−1^ RR, underlining the need for optimizing ventilator settings for each individual patient’s physiology. Typically, we encountered a problem where the expiratory volumes recurrently fell short of the inspiratory volumes, resulting in respiratory pattern where the excess inspired gas mixture was typically exhaled after every three or four inspirations. The communication of inspired gas mixture with the pneumothorax cavity and a small leakage from our spirometry hosing in the spirometry module, identified at a late stage may have contributed to this phenomenon.

We decided to omit the analysis of dead space ventilation and atelectatic lung tissue. It is probable that part of the gas mixture travels in the central spaces of the airways bypassing much of the dead space volume and obscuring the calculation of dead space ventilation percentage. The quantification of atelectasis is difficult, and in our setting atelectasis was largely secondary to pneumothoraces making it rather redundant.

In future, it would be worthwhile to examine the effects and performance of the CCSV protocol with a more sophisticated optimization aiming with a protocolized approach to reach 100% synchronization and lower tidal volumes of 2–3 ml/kg. This might reduce the number of pneumothoraces.

## Conclusions

Our study showed higher oxygenation of arterial blood and no difference in blood pressure levels in the CCSV protocol compared to manual bag-valve ventilation in a porcine model of experimental prolonged CA. The CCSV group did however have more pneumothoraces. Although the clinical relevance of this finding is unknown there needs to be further studies focusing on more optimized synchronization of compressions, and the use of lower levels of inspiratory pressure.

## Consent for publication

Not applicable.

## Availability of data and material

The datasets used and/or analysed during the current study are available from the corresponding author on reasonable request.

## CRediT authorship contribution statement

**Jukka Kopra:** Writing – review & editing, Writing – original draft, Visualization, Project administration, Methodology, Investigation, Formal analysis, Data curation, Conceptualization. **Lassi Mehtonen:** Writing – review & editing, Investigation, Data curation. **Merja Laitinen:** Writing – review & editing, Formal analysis. **Erik Litonius:** Writing – review & editing, Writing – original draft, Project administration, Methodology, Investigation, Funding acquisition, Conceptualization. **Oiva Arvola:** Writing – review & editing, Methodology, Investigation. **Robert Östman:** Writing – review & editing, Investigation. **Juho A. Heinonen:** Writing – review & editing, Methodology, Investigation. **Markus B. Skrifvars:** Writing – review & editing, Writing – original draft, Supervision, Project administration, Methodology, Investigation, Funding acquisition, Conceptualization. **Pirkka T. Pekkarinen:** Writing – review & editing, Writing – original draft, Supervision, Project administration, Methodology, Investigation, Conceptualization.

## Ethics approval

The study was approved by the Finnish National Animal Experiment Board (ESAVI-26974-2023). The study is reported in adherence to the ARRIVE guidelines.

## Funding

This study was supported by academic grants from Sigrid Juselius Stiftelse (Grant recipient MBS), Svenska Kulturfonden, Medicinska Understödsföreningen Liv och Hälsa and Finska Läkaresällskapet.

## Declaration of competing interest

The authors declare the following financial interests/personal relationships which may be considered as potential competing interests: MBS reports speaker fees from BARD Medical (Ireland). The other authors state that they have no conflicts of interests. Weinmann Emergency Medical Technology GmbH + Co provided the group with the MEDUMAT Standard^2^ ventilator free of charge, but did not otherwise provide funding for the research. MBS is part of the Resuscitation journal's Editorial Board.

## References

[1] Magliocca A., Olivari D., De Giorgio D. (2019). LUCAS versus manual chest compression during ambulance transport: a hemodynamic study in a porcine model of cardiac arrest. J Am Heart Assoc.

[2] Bekgöz B., Şan İ., Ergin M. (2020). Quality comparison of the manual chest compression and the mechanical chest compression during difficult transport conditions. J Emerg Med.

[3] Rezoagli E., Magliocca A., Grieco D.L., Bellani G., Ristagno G. (2022). Impact of lung structure on airway opening index during mechanical versus manual chest compressions in a porcine model of cardiac arrest. Respir Physiol Neurobiol.

[4] Doeleman L.C., Boomars R., Radstok A. (2024). Ventilation during cardiopulmonary resuscitation with mechanical chest compressions: How often are two insufflations being given during the 3-second ventilation pauses?. Resuscitation.

[5] Markstaller K., Karmrodt J., Doebrich M. (2002). Dynamic computed tomography: a novel technique to study lung aeration and atelectasis formation during experimental CPR. Resuscitation.

[6] Song S.R., Kim K.H., Park J.H., Song K.J., Do S.S. (2023). Association between prehospital airway type and oxygenation and ventilation in out-of-hospital cardiac arrest. Am J Emerg Med.

[7] Spindelboeck W., Gemes G., Strasser C. (2016). Arterial blood gases during and their dynamic changes after cardiopulmonary resuscitation: a prospective clinical study. Resuscitation.

[8] Spindelboeck W., Schindler O., Moser A. (2013). Increasing arterial oxygen partial pressure during cardiopulmonary resuscitation is associated with improved rates of hospital admission. Resuscitation.

[9] Nelskylä A., Skrifvars M.B., Ångerman S., Nurmi J. (2022). Incidence of hyperoxia and factors associated with cerebral oxygenation during cardiopulmonary resuscitation. Resuscitation.

[10] Bartos J.A., Clare Agdamag A., Kalra R. (2023). Supraglottic airway devices are associated with asphyxial physiology after prolonged CPR in patients with refractory Out-of-Hospital cardiac arrest presenting for extracorporeal cardiopulmonary resuscitation. Resuscitation.

[11] Shou B.L., Ong C.S., Premraj L. (2023). Arterial oxygen and carbon dioxide tension and acute brain injury in extracorporeal cardiopulmonary resuscitation patients: analysis of the extracorporeal life support organization registry. J Heart Lung Transplant.

[12] Vissers G., Soar J., Monsieurs K.G. (2017). Ventilation rate in adults with a tracheal tube during cardiopulmonary resuscitation: a systematic review. Resuscitation.

[13] Kill C., Manegold R.K., Fistera D., Risse J. (2024). Airway management and ventilation techniques in resuscitation during advanced life support: an update. J Anesth Analgesia Crit Care.

[14] Kill C., Hahn O., Dietz F. (2014). Mechanical ventilation during cardiopulmonary resuscitation with intermittent positive-pressure ventilation, bilevel ventilation, or chest compression synchronized ventilation in a pig model. Crit Care Med.

[15] Grauman S., Johansson J., Drevhammar T. (2021). Large variations of oxygen delivery in self-inflating resuscitation bags used for preoxygenation - a mechanical simulation. Scand J Trauma Resusc Emerg Med.

[16] Soar J., Böttiger B.W., Carli P. (2021). European Resuscitation Council Guidelines 2021: adult advanced life support. Resuscitation.

[17] Cordioli R.L., Brochard L., Suppan L. (2018). How ventilation is delivered during cardiopulmonary resuscitation: an international survey. Respir Care.

[18] Idris A.H., Aramendi Ecenarro E., Leroux B. (2023). Bag-valve-mask ventilation and survival from out-of-hospital cardiac arrest: a multicenter study. Circulation.

[19] Orlob S., Wittig J., Hobisch C. (2021). Reliability of mechanical ventilation during continuous chest compressions: a crossover study of transport ventilators in a human cadaver model of CPR. Scand J Trauma Resusc Emerg Med.

[20] Aufderheide T.P., Lurie K.G. (2004). Death by hyperventilation: a common and life-threatening problem during cardiopulmonary resuscitation. Crit Care Med.

[21] Aufderheide T.P., Sigurdsson G., Pirrallo R.G. (2004). Hyperventilation-induced hypotension during cardiopulmonary resuscitation. Circulation.

[22] Kill C., Galbas M., Neuhaus C. (2015). Chest compression synchronized ventilation versus intermitted positive pressure ventilation during cardiopulmonary resuscitation in a pig model. PLoS One.

[23] Xu J., Khan Z.U., Zhang M. (2022). The combination of chest compression synchronized ventilation and aortic balloon occlusion improve the outcomes of cardiopulmonary resuscitation in swine. Front Med (Lausanne).

[24] Renz M., Müller L., Herbst M. (2023). Analysis of cerebral Interleukin-6 and tumor necrosis factor alpha patterns following different ventilation strategies during cardiac arrest in pigs. PeerJ.

[25] Kopra J., Litonius E., Pekkarinen P.T. (2024). Oxygenation and ventilation during prolonged experimental cardiopulmonary resuscitation with either continuous or 30:2 compression-to-ventilation ratios together with 10 cmH20 positive end-expiratory pressure. Intensive Care Med Exp.

[26] Kopra J., Litonius E., Pekkarinen P.T. (2023). Ventilation during continuous compressions or at 30:2 compression-to-ventilation ratio results in similar arterial oxygen and carbon dioxide levels in an experimental model of prolonged cardiac arrest. Intensive Care Med Exp.

[27] Patata V., Caivano D., Porciello F. (2020). Pulmonary vein to pulmonary artery ratio in healthy and cardiomyopathic cats. J Vet Cardiol.

[28] Roels E., Fastrès A., Merveille A.C. (2021). The prevalence of pulmonary hypertension assessed using the pulmonary vein-to-right pulmonary artery ratio and its association with survival in West Highland white terriers with canine idiopathic pulmonary fibrosis. BMC Vet Res.

[29] Patata V., Caivano D., Porciello F. (2020). Pulmonary vein to pulmonary artery ratio in healthy and cardiomyopathic cats. J Vet Cardiol.

[30] Souza L.V.S., Zanon M., Souza A.S. (2017). “Pulmonary Vein Sign” for pulmonary embolism diagnosis in computed tomography angiography. Lung.

[31] Lee J., Chung J., Baek L., Je M., Choi J., Yoon J. (2022). Respiratory phase can affect pulmonary vein-to-pulmonary artery ratio measured with CT. Am J Vet Res.

[32] Kim M.S., Kim J., Seo M.W., Park C. (2024). Pulmonary-vein-to-pulmonary-artery ratio can be utilized to evaluate myxomatous mitral valve disease progression in dogs. Am J Vet Res.

[33] Renz M., Müllejans L., Riedel J. (2022). High PEEP Leveals during CPR improve ventilation without deleterious haemodynamic effects in pigs. J Clin Med.

[34] Ruemmler R., Ziebart A., Moellmann C. (2018). Ultra-low tidal volume ventilation-A novel and effective ventilation strategy during experimental cardiopulmonary resuscitation. Resuscitation.

