# Supplementary material for: Chest compression synchronized ventilation during prolonged experimental cardiopulmonary resuscitation improves oxygenation but may cause pneumothoraces
Source: Resusc Plus. 2025 Feb 28;22:100918. doi: 10.1016/j.resplu.2025.100918 (PMC11953954; doi:10.1016/j.resplu.2025.100918)
Supplement: Supplementary Data 2 [file mmc2.docx]

## Chest compression synchronized ventilation during prolonged experimental cardiopulmonary resuscitation improves oxygenation but may cause pneumothoraces

Kopra J. et al.

**Supplementary material 2:**

Spirometry data extracted from MEDUMAT Standard^2^ ventilator (Chest compression synchronized ventilation group only; n = 14).

|  | Median (IQR) |
| --- | --- |
| Respiratory rate (min ^-1^) | 86 (76 - 101) |
| Minute volume (litres) | 17.3 (10.0 - 20.8) |
| Tidal volume (ml) | 230 (110 - 290) |
| Peak pressure (cmH_2_O) | 62.3 (55.7 - 64.5) |
| Positive end-expiratory pressure (cmH_2_O) | 5.5 (3.1 - 9.7) |

cmH_2_O, centimetres of water; IQR, Interquartile range.
